# Microstructural Evolution and Mechanical Properties of a Ni-Based Alloy with High Boron Content for the Pre-Sintered Preform (PSP) Application

**DOI:** 10.3390/ma16237483

**Published:** 2023-12-02

**Authors:** Xiufang Gong, Yunsheng Wu, Zhenhuan Gao, Youbei Sun, Yingbo Guan, Xianjun Guan, Xuezhi Qin, Jieshan Hou, Lanzhang Zhou

**Affiliations:** 1 State Key Laboratory of Clean and Efficient Turbomachinery Power Equipment, Deyang 618000, China; 2 Dongfang Electric Corporation Dongfang Turbine Co. Ltd., Deyang 618000, China; 3 Shi-Changxu Innovation Center for Advanced Materials, Institute of Metal Research, Chinese Academy of Sciences, Shenyang 110016, China; xjguan@imr.ac.cn (X.G.); xzqin@imr.ac.cn (X.Q.); jshou@imr.ac.cn (J.H.); 4 CAS Key Laboratory of Nuclear Materials and Safety Assessment, Institute of Metal Research, Chinese Academy of Sciences, Shenyang 110016, China

**Keywords:** microstructural evolution, tensile strength, Ni-based alloy, high boron content, pre-sintered preform (PSP)

## Abstract

The pre-sintered preform (PSP) is an advanced technology for repairing the Ni-based superalloy blade in a turbine. In general, boron is added to the Ni-based superalloys in small quantities (<0.1 wt.%) to increase boundary strength and cohesivity. Despite this, the effect of high B content (>1.0 wt.%) on the microstructure evolution and mechanical properties in Ni-based superalloys for the PSP application is rarely studied. The variety, composition and evolution of the precipitates during solution heat treatment in the alloy with high B content were determined by EBSD, EPMA and SEM. The results indicate that Cr, W and Mo-rich M_5_B_3_ type borides precipitate from the matrix and its area fraction reaches up to about 8%. The area fraction of boride decreases with the prolonging of solution time and the increase of temperature higher than 1120 °C. The borides nearly disappear after solution treatment at 1160 °C for 2 h. The redissolution of boride and eutectic results in the formation of B-rich area with low incipient melting (about 1189 °C). It can bond metallurgically with the blade under the melting point of the blade, which decreases the precipitation of harmful phases of the blade after PSP repairing. The microhardness within the grain in the PSP work-blank first decreases (lower than 1160 °C) and then increases (higher than 1185 °C) with the increase of solution heat treatment temperature due to the dissolving and precipitation of borides. The tensile strength of the combination of PSP work-blank and Mar-M247 matrix at room temperature after solution treatment is related to the area fraction of boride, incipient melting and the cohesion between PSP work-blank and Mar-M247 matrix.

## 1. Introduction

The pre-sintered preform (PSP) technology can repair the large plane, curved surface and wide gap damage of nickel-based superalloy blade in turbines due to its high mechanical properties and no obvious harmful phases after repair when compared with traditional brazing and fusion welding. The high melting point powder (Mar-M247, IN738, GTD-111 etc.) and low melting point powder (the superalloy doping a large amount of B) are mixed evenly according to a certain proportion. The PSP work-blanks prepared by abrasive tools or hydraulic press are sintered in a vacuum furnace. The pre-sintered parts with a low melting point are machined to specific dimensions and then sintered in the vacuum furnace again to repair the large size defects.

The content of B in PSP is higher than 1.0 wt.%. However, boron is added to the Ni-based superalloys, in general, in small quantities (<0.1 wt.%) [1,2,3,4,5,6,7,8,9,10,11,12,13,14]. B can reside at the grain boundary and increase boundary strength and cohesivity, as well as decrease the agglomeration of M_23_C_6_ at the grain boundaries [1,2,15,16,17,18,19]. Therefore, B improves the mechanical properties of Ni-based superalloys, especially the creep performance [1,6,7,20,21,22]. D. Tytko et. al. [3] investigated the segregation behavior of B in alloy 617B by atom probe tomography. Enrichments of B at γ/M_23_C_6_ and γ′/M_23_C_6_ interfaces as well as at grain boundaries were detected, while no B enrichment was found at γ/γ′ interfaces. P. Kontis et. al. [1,2] investigated the effects of B on the microstructures and mechanical properties in a polycrystalline superalloy. Boron mainly resided in the form of M_5_B_3_ borides. Some boron segregation was found at the γ′/M_5_B_3_ interfaces. Nevertheless, other interfaces, such as γ/γ′, γ/M_5_B_3_, γ/MC and γ′/MC, showed no significant segregation. The addition of boron can improve the tensile ductility at 750 °C with a low strain rate. However, no increase in ductility was observed at a higher strain rate. Creep testing indicated that the optimum boron content in this alloy was 0.05 at.%. X. Li et. al. [6] investigated the effects of B on the stress rupture properties of a cast superalloy (K4750). The addition of B (0.01 wt.%) can increase the stress rupture properties, which resulted from the combination of boron segregation increased the cohesion of grain boundaries and granular M_23_C_6_ carbides suppressed the link-up and extension of micro-cracks. B. C. Yan et. al. [7] investigated the effect of boron additions on the transverse properties of a directionally solidified superalloy. The transverse creep strength increased dramatically when only 0.005 wt.% boron was added to the alloy.

Nevertheless, the effect of high B content (>1.0 wt.%) on the microstructures and mechanical properties in Ni-based superalloys is rarely investigated. It is necessary to clarify the microstructure evolution characteristic in Ni-based alloy with high B content. The results can offer the basal data for sintering and solution treatment technology in PSP.

## 2. Experiments

The high melting point powder and low melting point powder were manufactured by rotation electrode process. The compositions of high melting point powder were similar to IN738 alloy. The content of B in low melting point powder was higher than 2.5 wt.%. The compositions of the PSP work-blanks were 15.0Cr, 9.5Co, 5.5(Al+Ti+Nb), 4.2(Mo+W+Ta), 1.4B, 0.05C and Ni in balance (wt.%). The PSP work-blanks were pre-sintered by hydraulic press within a box, with the dimension of 100 × 50 × 8 mm, in a vacuum atmosphere. The pressure was derived from the metal block with the mass of 0.3 kg on the cover plate of the box. The pre-sintering temperature and time were 1190 °C~1245 °C and 5 min~30 min, respectively. The pre-sintered parts were sintered in the vacuum furnace a second time, to connect with the Mar-M247 matrix, at 1170 °C~1220 °C for about 1 h~4 h. The density of the PSP sintered part was about 8.0 g/cm^3^.

The microstructures were observed by TESCAN MAIA3 scanning electron microscope (SEM) with HKL-EBSD system. The element distributions of the investigated alloys were characterized using an EPMA-1610 electron probe micro analyzer (EPMA). The SEM and EPMA samples were ground, mechanically polished and then observed under the back scattering electron mode. The electron back-scattered diffraction (EBSD) samples were ground, mechanically polished and vibration polished for 12 h using a VIBROMET 2 vibratory polisher. EBSD investigation with a step size of 0.35 μm was carried out at 20 kV. Channel 5 software was employed for EBSD data processing.

The differential scanning calorimetry (DSC) was carried out using a STA449F3 ultra high temperature thermal analyzer to determine the phase-transition temperature and incipient melting point in the alloy with high B content. The calorimetry operated within the argon atmosphere. The temperature of the calorimeter was calibrated from the observed melting points of distilled water and ultra-pure materials (stearic acid, indium, tin and lead) at heating rate of 15 °C/min. Initially, the two empty pans were scanned to determine the calorimeter baseline. Finally, this was repeated with the investigated alloy. The cylindrical sample (3 mm in diameter and 2 mm in height) was used for the DSC test. The same heating rate of 15 °C/min and sample weight of 77.81 mg was used to obtain comparable results.

The solution heat treatment of the PSP work-blank was carried out using a muffle furnace to clarify the microstructural evolution. The solution heat treatment temperature and time are shown in Table 1 according to the results of DSC. The samples were placed in sealed glass tubes within an argon atmosphere before solution heat treatment in order to prevent oxidation. The microhardness within the grain in the PSP parts without and with solution heat treatment was conducted using a INNOVATEST FALCON 500. The loading force and time were 200 g and 13 s, respectively. At least four points were tested to obtain the average results of the microhardness. The tensile test of the combination of PSP work-blank and Mar-M247 matrix at room temperature without and with solution heat treatment were carried out on an AG-X 250 KN machine according to GB/T 228.1-2010. At least two samples were tested to obtain the average results of the tensile tests.

## 3. Results and Discussion

### 3.1. Microstructures

**Figure 1:**
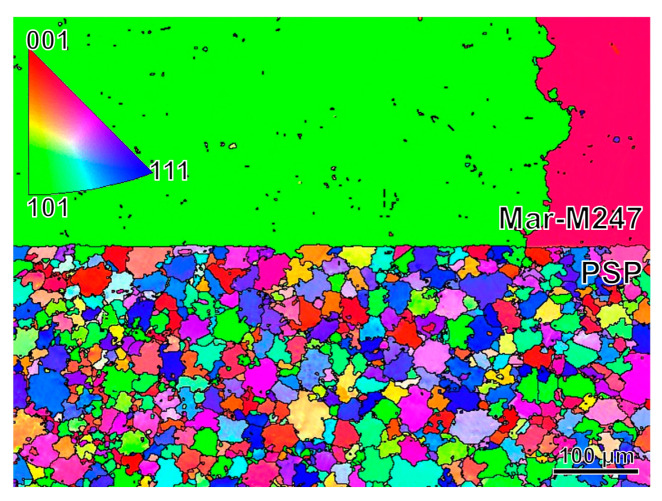
The microstructures of the PSP work-blank and Mar-M247 matrix near the interface observed by EBSD. The red, green and blue areas represent <001>, <101> and <111> grain orientation, respectively.

The microstructures of the PSP work-blank and Mar-M247 matrix near the interface are shown in Figure 1. The grain size of the matrix is coarsening, which is consistent with the microstructure of cast superalloy. The equiaxed grains with serrated boundaries appear in the PSP work-blank. The average grain size is about 40 μm. The red, green and blue areas in Figure 1 represent <001>, <101> and <111> grain orientation, respectively, as shown in the inset of Figure 1. The grain orientation is uniform and there is no obvious texture in the PSP work-blank. In addition, the interface between the PSP work-blank and the Mar-M247 matrix is straight. The large voids are not found near the interface.

**Figure 2:**
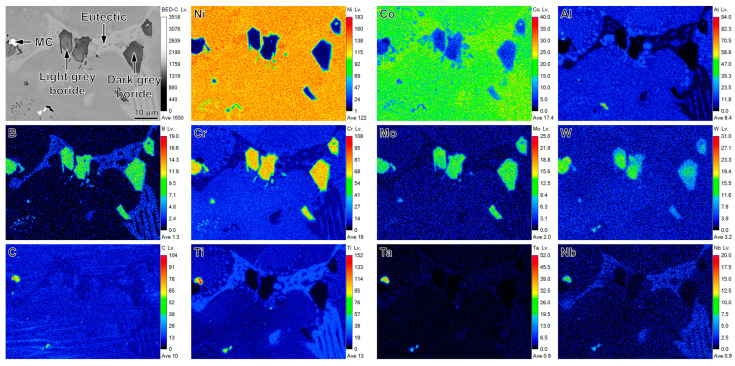
The main precipitates, including bright MC carbides (enriched C, Ti, Ta and Nb), light grey borides (enriched B, Cr, W and Mo), dark grey borides (enriched B, Cr, W and Mo) and eutectics in the PSP work-blank observed by EPMA.

Figure 2 exhibits the main precipitates of the PSP work-blank determined by EPMA. The results show that the small block bright precipitate, enriched with C, Ti, Ta and Nb, is determined as MC carbide. The light grey or dark grey precipitates in the B, Cr, W and Mo-rich large block are borides. In addition, the eutectic distributes around the borides. The quantitative analysis of the element content of the borides with different contrast is shown in Table 2 in order to determine the differentia of the borides. The borides are rich in Cr, W and Mo elements. The content of W in light grey boride (9.77 wt.%) is obviously higher than that in dark grey boride (5.78 wt.%), which results in the difference of the contrast of the borides. The borides with different contrasts possess a wrapping structure, as shown in Figure 2. The dark grey boride with lower W content surrounded the bright grey boride with higher W content. In general, the B atoms segregate at the interdendritic during solidification [1,6]. In addition, the bright grey borides with high W content nucleate in the interdendritic domain. W atoms are consumed with the growth of boride. However, W is a negative segregation element enriched in the dendritic core [23,24,25,26,27,28] and the diffusion rate of W atoms is slow due to the large atom size. The number of W atoms near the bright grey borides decreases with the growth of boride. Finally, the dark grey borides with low W content precipitate around the bright grey borides. In summary, the segregation characteristic and low diffusion rate of W atoms result in the precipitation of the borides with different contrast.

**Figure 3:**
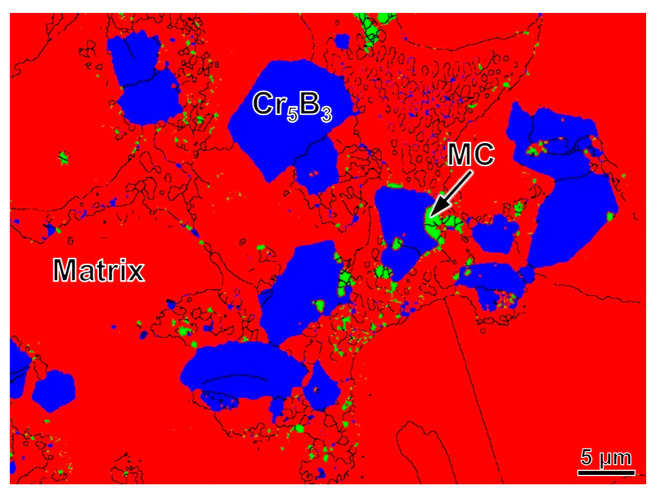
The variety of boride determined by EBSD. The red, green and blue areas represent matrix, MC carbide and Cr_5_B_3_ boride, respectively.

The variety of the boride was determined by phase analysis of EBSD, as shown in Figure 3. The red and green areas are matrix and MC carbide, respectively. The borides, marked by the blue area, are identified as Cr_5_B_3_. Parts of the Cr atoms are replaced by W and Mo atoms. The ratio of atom fraction of B and other elements is about 3:5 in Table 2, which verifies the result obtained by EBSD.

### 3.2. Solution Heat Treatment

**Figure 4:**
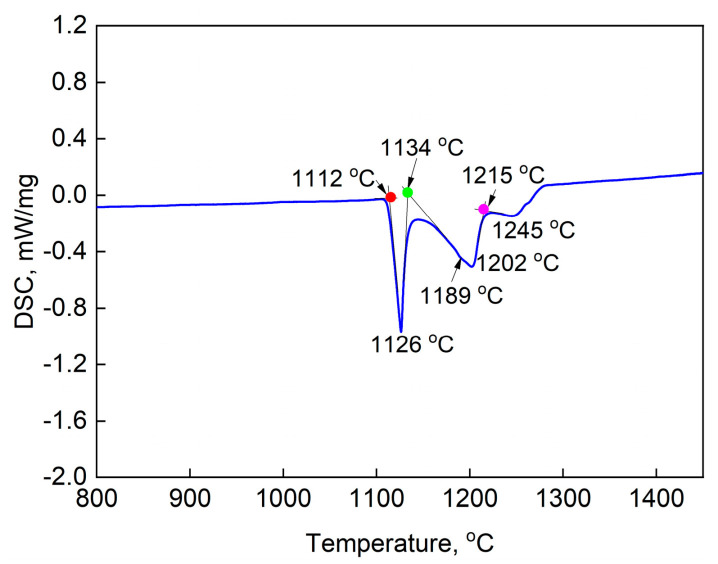
The DSC curve of PSP work-blank without solution heat treatment. The heating rate is 15 °C/min.

Figure 4 exhibits the DSC curve of PSP work-blank with solution heat treatment. Four endothermic peaks (1126 °C, 1189 °C, 1202 °C and 1245 °C) appear within 1100 °C~1250 °C during the heating process with the heating rate of 15 °C/min. The initial extrapolated temperatures of the endothermic peaks are 1112 °C, 1134 °C, 1189 °C and 1215 °C, respectively. The solution heat treatment temperature is determined according to the DSC results, as shown in Table 1. The solution heat treatment is conducted over the temperature range of 1100 °C~1220 °C at a 20 °C interval. The temperature range covers the four extrapolated temperatures of the endothermic peaks in DCS curve.

**Figure 5:**
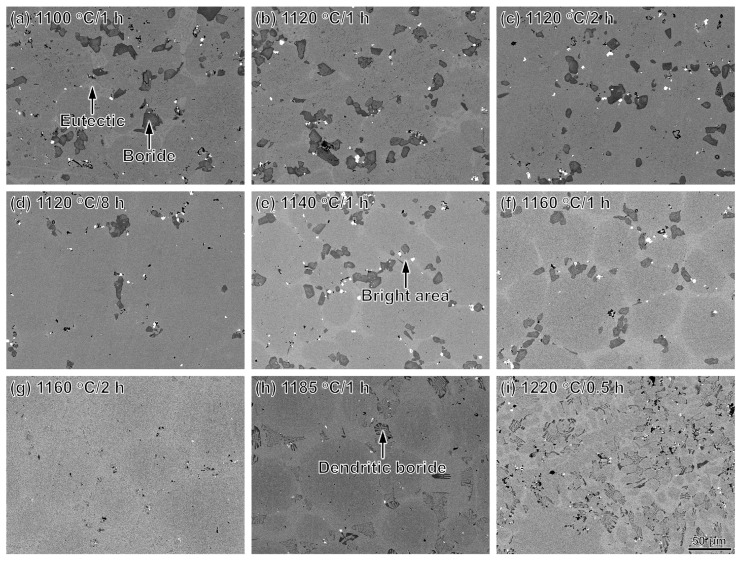
The microstructures of PSP work-blank after solution heat treatment at different temperatures and times in argon atmosphere with air cooling: (**a**) 1100 °C/1 h, (**b**) 1120 °C/1 h, (**c**) 1120 °C/2 h, (**d**) 1120 °C/8 h, (**e**) 1140 °C/1 h, (**f**) 1160 °C/1 h, (**g**) 1160 °C/2 h, (**h**) 1185 °C/1 h, (**i**) 1220 °C/0.5 h.

The microstructures of PSP work-blank after solution treatment are shown in Figure 5. The change of microstructures is not obvious after solution heat treatment at 1100 °C for 1 h (Figure 5a). The eutectics nearly disappear after solution treatment at 1120 °C for 2 h (Figure 5c), which indicates that the dissolving temperature of eutectic is 1112 °C~1125 °C, as shown in Figure 4.

**Figure 6:**
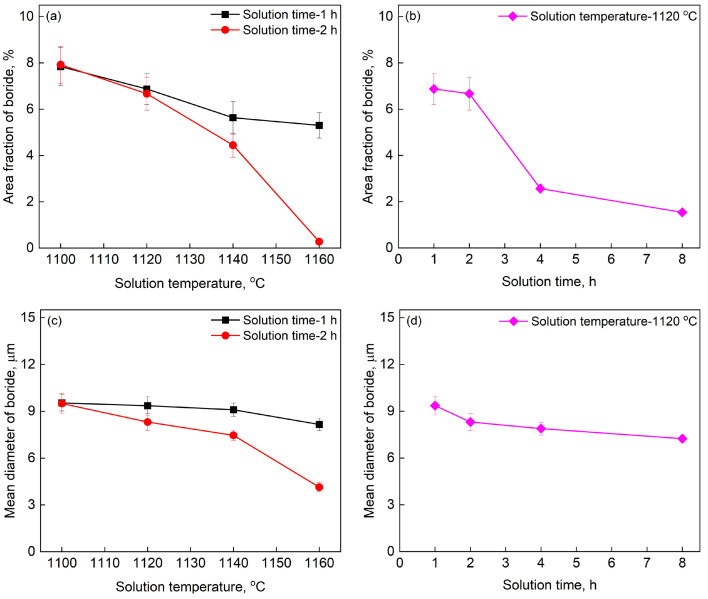
The area fraction and mean diameter of borides change with solution heat treatment temperature and time: (**a**) The change of boride area fraction with the solution temperature, (**b**) The change of boride area fraction with the solution time at 1120 °C, (**c**) The change of boride mean diameter with the solution temperature and (**d**) The change of boride mean diameter with the solution time at 1120 °C.

The area fraction and mean diameter of boride change with solution treatment temperature and time, as exhibited in Figure 5 and Figure 6. The area fraction of boride reaches up to 8% in the PSP work-blank, which is obviously higher than the superalloys with minor B. The area fraction of boride decreases with the increase of temperature and the borides nearly disappear after solution treatment at 1160 °C for 2 h, as shown in Figure 5c–g and Figure 6a. The area fraction of boride has no significant change after solution treatment at 1120 °C shorter than 2 h. However, it decreases rapidly after solution treatment for 4 h, as shown in Figure 6b. The area fraction of boride is only about 2% after solution treatment at 1120 °C/8 h. It can be seen that the boride can dissolve at 1120 °C, which is inconsistent with the DSC result (the dissolving temperature of boride could be 1134 °C according to Figure 4). The phenomenon resulted from the rapid heating rate during DSC. It results in a higher temperature of phase-transition compared with the actual value. In addition, the mean diameter of borides reaches 9 μm in the PSP work-blanks with high B content. It also decreases with the increase of temperature, as exhibited in Figure 6c. The size of borides reduces to about 3.5 μm after solution treatment at 1160 °C for 2 h. The mean diameter of borides decreases slowly with the prolonging of time at 1120 °C, as exhibited in Figure 6d.

The bright areas appear after solution treatment higher than 1140 °C at the domain with eutectic and boride, as shown in Figure 5e. The bright areas connect with each other with the increase of temperature and the prolonging of time, as shown in Figure 5f–h. The weight fraction of each element in the bright areas all fall in between the matrix and eutectic, as exhibited in Table 3. The bright areas contain higher B compared to the matrix. The redissolution of eutectic and boride in matrix results in the appearing of bright areas. The dendritic gray phase (B, Cr-rich boride according to EPMA) precipitates from the bright area after solution treatment at 1185 °C for 1 h, as exhibited in Figure 5h. It indicates that the incipient melting occurs within the bright area and the dendritic gray boride precipitates during solidification. This is consistent with the endothermic peaks at 1189 °C (Figure 4). The dendrite in matrix appears when the temperature is 1220 °C, as shown in Figure 5i. It indicates that the incipient melting occurs in matrix at this temperature, which is consistent with the endothermic peaks at 1215 °C in Figure 4.

### 3.3. Microhardness

**Figure 7:**
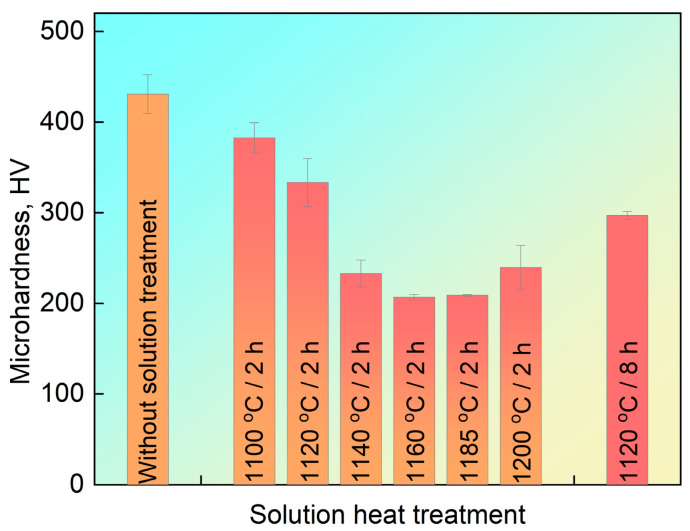
The microhardness within grains in the PSP work-blank without and with solution heat treatment.

The microhardness within grains in the PSP work-blank without and with solution heat treatment is shown in Figure 7. The microhardness decreases with the increase of solution temperature lower than 1160 °C. The precipitates (eutectics and borides) dissolve during solution heat treatment and the area fraction or mean diameter of borides both decrease with the increase of temperature, as exhibited in Figure 6a,c, which decreases the microhardness within grains. The dendritic borides precipitate after solution treatment higher than 1185 °C as shown in Figure 5h. The content of dendritic borides increases with the increase of solution heat treatment temperature higher than 1185 °C. Therefore, the microhardness increases with the increase of solution temperature at 1185 °C and 1200 °C. In addition, the area fraction and mean diameter of borides also decrease with the prolonging of solution heat treatment time, as shown in Figure 5c,d and Figure 6b,d. Therefore, the microhardness after solution heat treatment at 1120 °C for 8 h is lower than that for 2 h as shown in Figure 7.

### 3.4. Tensile Strength

**Figure 8:**
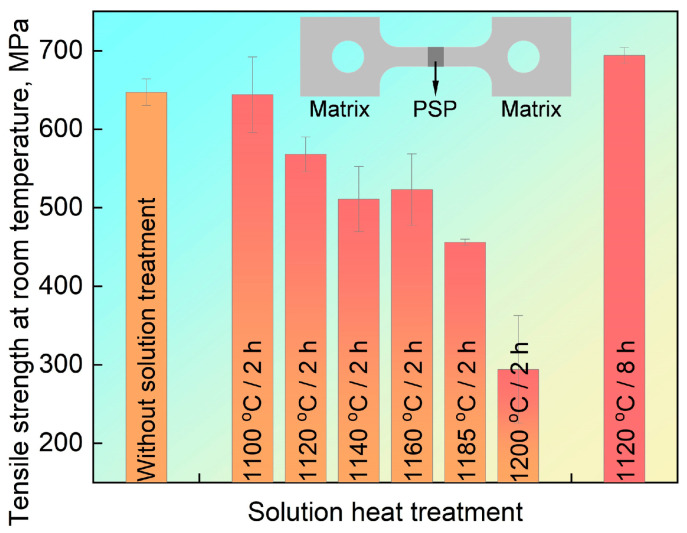
The stripe tensile sample and tensile strength at room temperature without and with solution heat treatment.

The tensile strength of the combination between PSP work-blank and Mar-M247 matrix at room temperature without and with solution heat treatment is shown in Figure 8. The stripe tensile sample is shown as the inset in Figure 8. The tensile strength of the combination alloy after solution heat treatment at 1100 °C/2 h is 644 MPa, which is similar to that without solution heat treatment (647 MPa). The tensile strength significantly decreases with the increase of solution temperature. It decreases to 294 MPa after solution heat treatment at 1200 °C/2 h. However, the tensile strength after solution heat treatment at 1120 °C/8 h (694 MPa) is higher than that without solution heat treatment.

It can be speculated that the tensile strength is related to the area fraction of boride, incipient melting and the cohesion between PSP work-blank and Mar-M247 matrix. First of all, the boride and carbide can hinder dislocation motion [1,29,30], which increase the tensile strength. The area fraction of boride has little change after solution treatment at 1100 °C/2 h as shown in Figure 5a. Therefore, its tensile strength is similar to that without solution treatment. The borides dissolve when the solution temperature is higher than 1120 °C. The tensile strength reduces with the decrease of borides. The tensile strength decreases to 511 MPa after solution heat treatment at 1140 °C for 2h. Then, the cohesion between PSP work-blank and Mar-M247 matrix can also influence the tensile strength. As shown in Figure 5c,d and Figure 6b, the area fraction of boride after solution treatment at 1120 °C/2 h is higher than that at 1120 °C/8 h. However, the tensile strength after solution treatment at 1120 °C/2 h (568 MPa) is lower than that at 1120 °C/8 h (694 MPa). It results from the increasing of cohesion between PSP work-blank and Mar-M247 matrix with the prolonging of solution time. In addition, the cohesion can also increase with the increase of temperature due to the higher diffusion rate. Hence, the tensile strength after solution treatment at 1160 °C/2 h (523 MPa) is higher than that at 1140 °C/2 h (511 MPa). Finally, the incipient melting of the alloy can not be neglected. The cohesion between PSP work-blank and Mar-M247 matrix increases with the increase of temperature, which increases the tensile strength further after solution treatment higher than 1160 °C. However, it is not the case as shown in Figure 8. The incipient melting occurs when the temperature is higher than 1185 °C. It will lead to the precipitation of the dendritic gray boride. The dendritic borides become crack initiation sites due to the stress concentration near the tips of dendritic borides. Therefore, the tensile strength decreases after solution temperature higher than 1185 °C. It decreases to 456 MPa and 294 MPa after solution heat treatment at 1185 °C/2 h and 1200 °C/2 h, respectively.

## 4. Conclusions

The microstructure characteristic, microstructure evolution after solution heat treatment at different temperature and time and its effect on microhardness as well as the tensile strength at room temperature of PSP work-blank were investigated. The following conclusions can be drawn from the research:

(1) Cr, W and Mo-rich M_5_B_3_ type borides precipitate from the matrix and its area fraction reaches up to about 8% in the PSP work-blank with high B content (>1.0 wt.%).
(2) The area fraction of boride decreases with the prolonging of solution time and the increase of temperature higher than 1120 °C. The borides nearly disappear after solution treatment at 1160 °C for 2 h.
(3) The redissolution of boride and eutectic results in the formation of B-rich bright area with low incipient melting (about 1189 °C). It bonds metallurgically with blade under the melting point of blade, which decreases the precipitation of harmful phases and increase the mechanical properties of the blade after PSP repairing.
(4) The microhardness within the grain in the PSP work blank decreases with the increase of solution temperature lower than 1160 °C due to the dissolving of borides. The dendritic borides precipitate after solution treatment higher than 1185 °C, which increases the microhardness.
(5) The tensile strength of the combination between PSP work-blank and Mar-M247 matrix at room temperature after solution treatment is related to the area fraction of boride, incipient melting and the cohesion between PSP work-blank and Mar-M247 matrix. The tensile strength reduces with the decrease of borides due to the block borides acting as obstacles to the dislocation motion. The cohesion between PSP work-blank and Mar-M247 matrix increases with the prolonging of solution time and the increase of solution temperature, which can increase the tensile strength. The dendritic borides precipitating after incipient melting will lead to the stress concentration and decrease the tensile strength.

## Figures and Tables

**Table 1:** The solution heat treatment temperature and time for the PSP work-blank.

Temperature/°C	1100	1120	1140	1160	1185	1200	1220
Time/h	1, 2, 4	1, 2, 4, 8	1, 2	1, 2	1, 2	1, 2	0.5

**Table 2:** The quantitative analysis of the element contents (weight fraction and atom fraction) in light grey boride and dark grey boride. The content of W in light grey boride is higher than that in dark grey boride.

Elements	Light Grey Boride		Dark Grey Boride	
	wt.%	at.%	wt.%	at.%
B	10.45	38.37	10.65	38.16
Cr	68.14	52.92	69.63	52.78
W	9.77	2.14	5.78	1.24
Mo	5.05	2.12	5.55	2.28
Ni	3.78	2.59	4.66	3.11
Co	2.44	1.67	3.31	2.21
Ti	0.16	0.13	0.19	0.16
Ta	0.20	0.04	0.20	0.04
Nb	0.01	0.00	0.03	0.01
Al	0.00	0.00	0.00	0.00

**Table 3:** The quantitative analysis of the element content (weight fraction and atom fraction) in eutectic, matrix and bright area. The weight fraction of each element in the bright areas all fall in between the matrix and eutectic.

Elements	Eutectic in Figure 5a		Matrix in Figure 5a		Bright Area in Figure 5e	
	wt.%	at.%	wt.%	at.%	wt.%	at.%
B	4.34	19.83	0.00	0.00	2.08	10.32
Cr	4.35	4.20	9.81	11.33	6.32	6.63
W	0.63	1.10	4.47	1.46	1.39	0.41
Mo	0.29	0.15	0.54	0.34	0.53	0.30
Ni	67.80	57.70	70.75	72.04	69.29	64.08
Co	11.72	9.98	8.61	8.77	11.55	10.68
Ti	4.48	4.69	2.51	3.14	3.94	4.48
Ta	3.62	3.62	2.00	0.66	2.97	0.90
Nb	2.18	1.18	0.40	0.26	1.21	0.71
Al	0.59	0.17	0.90	2.00	0.74	1.50

## Data Availability

Data are contained within the article.
